# Characteristics of unit-level patient safety culture in hospitals in Japan: a cross-sectional study

**DOI:** 10.1186/s12913-014-0508-2

**Published:** 2014-10-22

**Authors:** Shigeru Fujita, Kanako Seto, Takefumi Kitazawa, Kunichika Matsumoto, Tomonori Hasegawa

**Affiliations:** Department of Social Medicine, Toho University School of Medicine, 5-21-16 Omorinishi, Ota-ku, Tokyo 143-8540 Japan

**Keywords:** Patient safety, Organisational culture, Safety management, Attitude of health personnel, Patient safety culture

## Abstract

**Background:**

Patient safety culture (PSC) has an important role in determining safety and quality in healthcare. Currently, little is known about the status of unit-level PSC in hospitals in Japan. To develop appropriate strategies, characteristics of unit-level PSC should be investigated. Work units may be classified according to the characteristics of PSC, and common problems and appropriate strategies may be identified for each work unit category. This study aimed to clarify the characteristics of unit-level PSC in hospitals in Japan.

**Methods:**

In 2012, a cross-sectional study was conducted at 18 hospitals in Japan. The Hospital Survey on Patient Safety Culture questionnaire, developed by the United States Agency for Healthcare Research and Quality, was distributed to all healthcare workers (n =12,076). Percent positive scores for 12 PSC sub-dimensions were calculated for each unit, and cluster analysis was used to categorise the units according to the percent positive scores. A generalised linear mixed model (GLMM) was used to analyse the results of the cluster analysis, and odds ratios (ORs) for categorisation as high-PSC units were calculated for each unit type.

**Results:**

A total of 9,124 respondents (75.6%) completed the questionnaire, and valid data from 8,700 respondents (72.0%) were analysed. There were 440 units in the 18 hospitals. According to the percent positive scores for the 12 sub-dimensions, the 440 units were classified into 2 clusters: high-PSC units (n =184) and low-PSC units (n =256). Percent positive scores for all PSC sub-dimensions for high-PSC units were significantly higher than those for low-PSC units. The GLMM revealed that the combined unit type of ‘Obstetrics and gynaecology ward, perinatal ward or neonatal intensive care unit’ was significantly more likely to be categorised as high-PSC units (OR =9.7), and ‘Long-term care ward’ (OR =0.2), ‘Rehabilitation unit’ (OR =0.2) and ‘Administration unit’ (OR =0.3) were significantly less likely to be categorised as high-PSC units.

**Conclusions:**

Our study findings demonstrate that PSC varies considerably among different unit types in hospitals in Japan. Factors contributing to low PSC should be identified and possible measures for improving PSC should be developed and initiated.

**Electronic supplementary material:**

The online version of this article (doi:10.1186/s12913-014-0508-2) contains supplementary material, which is available to authorized users.

## Background

The concept of patient safety culture (PSC) has been increasingly used for improving safety and quality in healthcare. PSC is defined as the product of individual and group values, attitudes, perceptions, competencies and patterns of behaviour, which determine the commitment to, and the style and proficiency of, an organisation’s health and safety management [1]. Positive PSC has been reported to be associated with enhanced patient safety [2,3].

Several questionnaires have been developed to measure PSC [4]. The Hospital Survey of Patient Safety Culture (HSOPSC) questionnaire was developed by the United States Agency for Healthcare Research and Quality (AHRQ), and it has been widely used to measure institutional- and national-level PSC and to examine the effectiveness of strategies designed to improve patient safety and PSC [5,6]. Some previous studies have suggested the importance of measuring work unit-level PSC, and recommended that strategies for improving PSC should be tailored to individual work units [5,7]. Although some studies have identified work units with low PSC, such as intensive care units and emergency departments, little is known about the current status of unit-level PSC in Japan [8,9]. Understanding the characteristics of unit-level PSC could help to develop appropriate strategies to improve PSC. Work units could be classified according to the characteristics of PSC, common problems could be identified and appropriate strategies developed to meet the needs of each work unit.

This study aimed to investigate the characteristics and status of unit-level PSC in hospitals in Japan.

## Methods

In 2012, a cross-sectional study was conducted in 18 hospitals in Japan. All hospitals participated voluntarily in the study. Eight of the 18 hospitals had less than 200 beds, seven had between 200 and 499 beds and three had 500 or more beds. Three of the 18 hospitals were long-term care hospitals, three were mixed-care hospitals and 12 were acute care hospitals. Questionnaires were distributed to all healthcare workers (n =12,076), completed anonymously and returned to a collection box in a provided envelope. Details of the participating hospitals are shown in Additional file 1.

The ethics committee of the Toho University School of Medicine approved the study.

### Questionnaire

**Figure 1:**
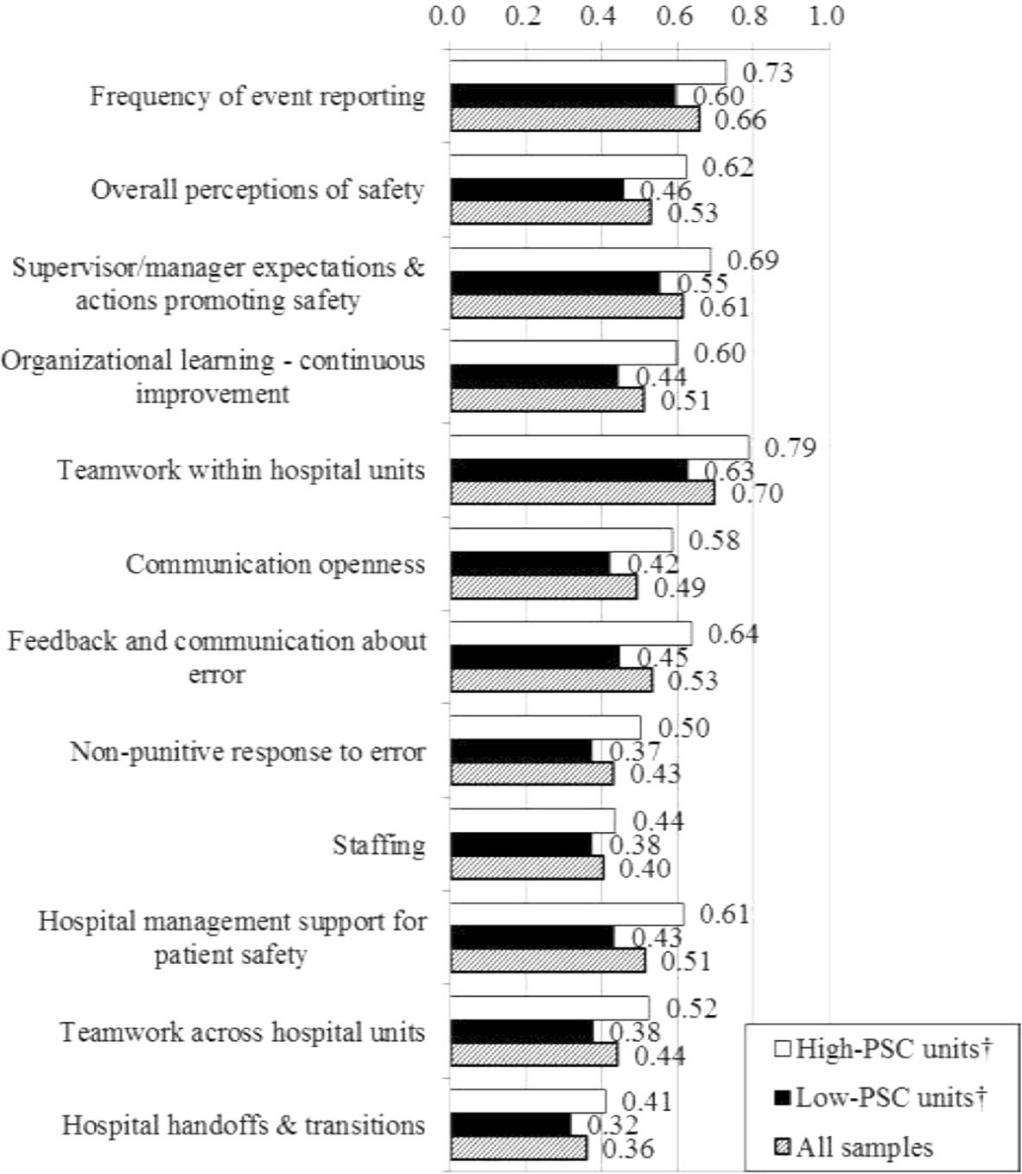
**Percent positive scores for the two clusters.** PSC: Patient safety culture ^†^All pairs of percent positive scores for high-PSC units and low-PSC units were significantly different (P <0.01).

To evaluate PSC, we used the HSOPSC questionnaire developed by the AHRQ [1]. The questionnaire includes 44 items to measure 12 PSC sub-dimensions (Figure 1), and 6 items to obtain background information of the respondent. The items to assess PSC use Likert scales with 5-point response options for agreement (from 1: ‘Strongly disagree’ to 5: ‘Strongly agree’) and frequency (from 1: ‘Never’ to 5: ‘Always’). Background information includes gender, current profession and working hours per week. The name of the respondent’s unit is recorded in an open-ended description section.

### Data analyses

Questionnaires were considered incomplete and excluded from analysis if the respondent answered less than one entire unit of the survey, less than half of the items throughout the entire survey or every item in the same way.

For each sub-dimension of PSC, the proportion of positive responses (percent positive score) was calculated for every respondent based on the AHRQ instructions. The proportion ranged from 0 to 1 with higher scores indicating a more positive PSC [1]. Percent positive scores for each unit were calculated by taking the mean of percent positive scores for all respondents who belonged to that unit. The units were categorised into 16 types such as ‘General ward’, ‘Outpatient unit’, ‘Pharmaceutical unit’ and ‘Operating room’. Obstetrics and gynaecology wards, perinatal wards and neonatal intensive care units (NICU) were combined to form one unit type because those units are generally part of a single nursing entity with shared facilities and resources. A separate ‘Physicians’ unit’ was created because, in Japan, most physicians are directly employed by hospitals and their responsibilities are not limited to just one ward. In a typical hospital organization chart, there are some physicians’ units and most physicians belong to one of the physicians’ units in Japan [10]. ‘Administration unit’ includes accountancy sections, personnel sections, supplies sections or general sections in which most of the staff don’t provide direct care. Units with less than five respondents were categorised as ‘Others’ because it was difficult to evaluate the PSC of units with small staff numbers.

Hierarchical cluster analysis based on the squared Euclidean distance and Ward’s method was used to categorise the units according to the percent positive scores for the 12 sub-dimensions. The percent positive scores of the 12 sub-dimensions were compared between the two clusters using *t* tests. A generalised linear mixed model (GLMM) was used to analyse the results of the cluster analysis, and odds ratios (ORs) for categorisation as high-PSC units were calculated according to the unit type. In addition, ORs for the contribution of sub-dimensions to categorisation as a high-PSC unit were computed from the GLMM analysis for each unit type. The difference between hospitals was treated as a random effect in the GLMM. A chi-square test was performed to compare categorical variables. Statistical analyses were performed using SPSS 19.0 (SPSS Inc.; Chicago, IL, USA).

## Results

A total of 9,124 respondents (75.6%) completed the questionnaire, and valid data from 8,700 respondents (72.0%) were analysed. Each participating hospital had between 7 and 77 units, and there were 440 units in the 18 hospitals. Each unit had 5–115 respondents (median =17.5). Among the respondents, 9.2% were physicians, 46.4% were nurses, 14.4% were administrative workers and 30.0% had other roles (Table 1). The professional composition of each unit type is shown in Additional file 2.

**Table 1:** **Characteristics of respondents and units**

		**Number of respondents**		**Number of units**	
		**n**	**(%)**	**n**	**(%)**
Location of hospital	Urban area	5,999	(69.0)	291	(66.1)
	Rural area	2,701	(31.0)	149	(33.9)
Type of hospital	Acute care	7,603	(87.4)	384	(87.3)
	Long-term care or mixed-care	1,097	(12.6)	56	(12.7)
Number of beds	<200 beds	1,359	(15.6)	75	(17.0)
	200–499 beds	3,715	(42.7)	199	(45.2)
	≥500 beds	3,626	(41.7)	166	(37.7)
Unit type	General ward	2,279	(26.2)	93	(21.1)
	Physicians’ unit^†^	777	(8.9)	67	(15.2)
	Administration unit	1,017	(11.7)	57	(13.0)
	Outpatient unit	548	(6.3)	19	(4.3)
	Clinical laboratory or physiology unit	237	(2.7)	17	(3.9)
	Long-term care ward	364	(4.2)	17	(3.9)
	Dietary unit	250	(2.9)	16	(3.6)
	Rehabilitation unit	379	(4.4)	16	(3.6)
	Pharmaceutical unit	214	(2.5)	13	(3.0)
	Critical care centre, ICU or CCU	364	(4.2)	11	(2.5)
	Obstetrics and gynaecology ward, perinatal ward or NICU	330	(3.8)	11	(2.5)
	Clinical radiology unit	181	(2.1)	10	(2.3)
	Operating room	232	(2.7)	10	(2.3)
	Dialysis unit	92	(1.1)	6	(1.4)
	Paediatric ward	177	(2.0)	6	(1.4)
	Others	1,259	(14.5)	71	(16.1)
Profession	Nurse	4,039	(46.4)	-	
	Clerk	1,253	(14.4)	-	
	Physician	807	(9.3)	-	
	Nursing aide or care worker	639	(7.3)	-	
	Technician	532	(6.1)	-	
	Therapist	376	(4.3)	-	
	Dietician or cook	260	(3.0)	-	
	Pharmacist	183	(2.1)	-	
	Others	550	(6.3)	-	
	Not reported	61	(0.7)		
Gender	Male	2,052	(23.6)	-	
	Female	6,338	(72.9)	-	
	Not reported	310	(3.6)	-	
Total		8,700		440	

CCU: Cardiac Care Unit; ICU: Intensive Care Unit; NICU: Neonatal Intensive Care Unit.

^†^Physicians do not usually work in a single unit, but are included in the physicians’ unit.

According to the percent positive scores for the 12 sub-dimensions, the 440 units were classified into two clusters: high-PSC units (n =184) and low-PSC units (n =256) (Table 2). Percent positive scores for all PSC sub-dimensions were significantly higher for high-PSC units than for low-PSC units (Figure 1).

**Table 2:** **Classification of the two clusters and odds ratios for categorisation as a high-PSC unit**

		**Number of units**			**The odds ratios for classification as high-PSC units** ^**‡**^		
		**Cluster 1: High-PSC units**	**Cluster 2: Low-PSC units**	**Total**	**Odds ratio**	**95% CI**	**P-value**
		**(n =184)**	**(n =256)**	**(n =440)**			
Location of hospital	Urban area	128	163	291	1.00		
	Rural area	56	93	149	1.12	(0.44–2.89)	0.81
Type of hospital	Acute care	170	214	384	1.00		
	Long-term care or mixed-care	14	42	56	0.88	(0.20–3.98)	0.87
Number of beds	<200 beds	22	53	75	1.00		
	200–500 beds	79	120	199	0.81	(0.19–3.45)	0.77
	≥500 beds	83	83	166	1.20	(0.22–6.71)	0.83
Unit type	General ward	46	47	93	1.00		
	**Obstetrics and gynaecology ward, perinatal ward or NICU**	10	1	11	**9.71**	**(1.17–80.74)**	**0.04** ^**†**^
	Operating room	6	4	10	1.76	(0.45–6.87)	0.42
	Clinical laboratory or physiology unit	10	7	17	1.47	(0.50–4.31)	0.48
	Outpatient unit	11	8	19	1.44	(0.52–4.00)	0.48
	Physicians’ unit^¶^	36	31	67	1.16	(0.60–2.26)	0.66
	Critical care centre, ICU or CCU	6	5	11	1.10	(0.31–3.98)	0.88
	Paediatric ward	3	3	6	1.06	(0.20–5.72)	0.94
	Dietary unit	6	10	16	0.66	(0.21–2.07)	0.47
	Others	26	45	71	0.61	(0.32–1.18)	0.14
	Dialysis unit	2	4	6	0.57	(0.10–3.36)	0.53
	Pharmaceutical unit	4	9	13	0.49	(0.14–1.77)	0.28
	Clinical radiology unit	3	7	10	0.48	(0.11–2.02)	0.32
	**Administration unit**	11	46	57	**0.26**	**(0.12–0.56)**	**<0.01** ^**†**^
	**Rehabilitation unit**	2	14	16	**0.16**	**(0.03–0.79)**	**0.02** ^**†**^
	**Long-term care ward**	2	15	17	**0.15**	**(0.03–0.83)**	**0.03** ^**†**^

PSC: Patient Safety Culture; CI: Confidence Interval; NICU: Neonatal Intensive Care Unit; ICU: Intensive Care Unit; CCU: Coronary Care Unit.

^¶^Physicians do not usually work in a single section, but are included in the physicians’ unit.

^‡^Results of the generalised linear mixed model (GLMM) using unit-level data. The reference category was low-PSC units. The differences between hospitals were included in the GLMM as random effects. The predictive value of classification using the GLMM was 68.6%.

^†^P <0.05.

The results of the GLMM showed that the combined unit type of ‘Obstetrics and gynaecology ward, perinatal ward or NICU’ were significantly more likely to be categorised as high-PSC units (OR =9.7), and ‘Administration unit’ (OR =0.3), ‘Rehabilitation unit’ (OR =0.2) and ‘Long-term care ward’ (OR =0.2) were significantly less likely to be categorised as high-PSC units (Table 2).

Percent positive scores of PSC sub-dimensions for each unit type are shown in Additional file 3. Percent positive scores for ‘Organisational learning - continuous improvement’ and ‘Hospital management support for patient safety’ were highest in ‘Obstetrics and gynaecology ward, perinatal ward or NICU’. Percent positive scores for ‘Frequency of events reported’ and ‘Organisational learning - continuous improvement’ were lowest in ‘Administration unit’, and percent positive scores for ‘Communication openness’, ‘Teamwork across hospital units’ and ‘Hospital handoffs and transitions’ were lowest in ‘Rehabilitation unit’. Percent positive scores for ‘Overall perceptions of patient safety’, ‘Communication openness’, ‘Staffing’, ‘Hospital management support for patient safety’ and ‘Patient safety grade’ were lowest in ‘Long-term care ward’.

Among all sub-dimensions, ‘Teamwork within hospital units’ (OR =1.8) had the biggest influence on whether or not a unit would be categorised as a high-PSC unit (Table 3). By unit type, the most important sub-dimension for categorising a unit as high-PSC was ‘Supervisor/manager expectations and actions promoting safety’ in ‘General ward’ (OR =2.3) and ‘Long-term care ward’ (OR = 27.08), ‘Non-punitive response to error’ in ‘Administration unit’ (OR =3.8), ‘Hospital handoffs and transitions’ in ‘Physicians’ unit’ (OR =2.2), ‘Feedback and communication about error’ in ‘Outpatient unit’ (OR =6.4) and ‘Staffing’ in ‘Critical care centre, intensive care unit (ICU) or coronary care unit (CCU)’ (OR = 9.28). The GLMMs for the other unit types did not reach convergence because of the insufficient sample size.

**Table 3:** **Key sub-dimensions for categorisation as a high-PSC unit** ^**‡**^

**Sub-dimensions**	**All respondents**			**General ward**			**Administration unit**			**Physicians’ unit** ^**¶**^			**Outpatient unit**			**Long-term care ward**			**Critical care centre, ICU or CCU**		
	**(n =8,700)**			**(n =2,279)**			**(n =1,017)**			**(n =777)**			**(n =548)**			**(n =364)**			**(n =364)**		
	**Odds ratio**	**95% CI**	**P**	**Odds ratio**	**95% CI**	**P**	**Odds ratio**	**95% CI**	**P**	**Odds ratio**	**95% CI**	**P**	**Odds ratio**	**95% CI**	**P**	**Odds ratio**	**95% CI**	**P**	**Odds ratio**	**95% CI**	**P**
Frequency of events reported	**1.36**	**(1.19–1.56)**	**<0.01** ^**†**^	1.19	(0.87–1.64)	0.27	1.41	(0.68–2.91)	0.35	0.81	(0.50–1.31)	0.39	0.47	(0.15–1.46)	0.19	0.70	(0.18-2.71)	0.61	1.10	(0.46-2.65)	0.83
Overall perceptions of patient safety	**1.39**	**(1.14–1.70)**	**<0.01** ^**†**^	1.52	(0.97–2.39)	0.07	1.61	(0.55–4.73)	0.39	1.30	(0.65–2.61)	0.46	0.78	(0.16–3.77)	0.76	0.50	(0.04-5.80)	0.58	**4.57**	**(1.36-15.32)**	**0.01** ^**†**^
Supervisor/manager expectations and actions promoting safety	**1.54**	**(1.26–1.88)**	**<0.01** ^**†**^	**2.30**	**(1.45–3.64)**	**<0.01** ^**†**^	0.63	(0.20–1.97)	0.42	**2.13**	**(1.06–4.27)**	**0.03** ^**†**^	0.68	(0.15–3.08)	0.61	**27.08**	**(2.74-267.32)**	**0.01** ^**†**^	1.71	(0.53-5.48)	0.37
Organisational learning - continuous improvement	1.20	(1.00–1.43)	0.05	1.21	(0.81–1.82)	0.36	1.69	(0.66–4.32)	0.27	0.79	(0.42–1.50)	0.47	1.21	(0.30–4.80)	0.79	**16.64**	**(1.41-196.24)**	**0.03** ^**†**^	0.83	(0.27-2.59)	0.75
Teamwork within hospital units	**1.79**	**(1.49–2.16)**	**<0.01** ^**†**^	**2.12**	**(1.37–3.29)**	**<0.01** ^**†**^	2.98	(0.99–9.00)	0.05	1.59	(0.78–3.24)	0.20	1.47	(0.37–5.91)	0.58	0.27	(0.04-2.04)	0.21	1.79	(0.58-5.54)	0.31
Communication openness	1.16	(0.97–1.39)	0.10	0.89	(0.59–1.34)	0.58	1.42	(0.56–3.60)	0.46	1.15	(0.61–2.17)	0.68	0.58	(0.15–2.21)	0.42	1.77	(0.31-10.29)	0.52	0.32	(0.1-1.04)	0.06
Feedback and communication about error	**1.47**	**(1.23–1.75)**	**<0.01** ^**†**^	**1.62**	**(1.07–2.45)**	**0.02** ^**†**^	2.05	(0.80–5.31)	0.14	1.81	(0.95–3.46)	0.07	**6.35**	**(1.55–26.09)**	**0.01** ^**†**^	1.02	(0.14-7.53)	0.98	**4.75**	**(1.61-13.98)**	**0.01** ^**†**^
Non-punitive response to error	**1.20**	**(1.02–1.41)**	**0.03** ^**†**^	**1.66**	**(1.13–2.43)**	**0.01** ^**†**^	**3.81**	**(1.61–8.99)**	**<0.01** ^**†**^	0.83	(0.47–1.48)	0.52	2.73	(0.70–10.57)	0.15	0.43	(0.09-2.14)	0.30	0.89	(0.33-2.42)	0.82
Staffing	**1.32**	**(1.07–1.62)**	**0.01** ^**†**^	**2.02**	**(1.19–3.43)**	**0.01** ^**†**^	0.37	(0.13–1.10)	0.07	1.29	(0.61–2.73)	0.50	**4.72**	**(1.01–22.21)**	**0.05** ^**†**^	1.30	(0.14-12.23)	0.82	**9.28**	**(2.24-38.37)**	**<0.01** ^**†**^
Hospital management support for patient safety	**1.50**	**(1.25–1.80)**	**<0.01** ^**†**^	1.35	(0.90–2.04)	0.15	1.58	(0.61–4.13)	0.35	1.94	(0.99–3.79)	0.05	0.61	(0.15–2.45)	0.48	0.99	(0.15-6.72)	0.99	1.23	(0.39-3.93)	0.72
Teamwork across hospital units	1.08	(0.88–1.32)	0.47	0.65	(0.41–1.04)	0.07	1.70	(0.61–4.75)	0.31	1.14	(0.57–2.27)	0.71	1.67	(0.36–7.83)	0.52	1.28	(0.18-9.23)	0.81	1.34	(0.39-4.6)	0.64
Hospital handoffs and transitions	**0.72**	**(0.61–0.86)**	**<0.01** ^**†**^	0.71	(0.48–1.05)	0.09	0.47	(0.19–1.15)	0.10	**2.18**	**(1.18–4.05)**	**0.01** ^**†**^	0.62	(0.15–2.57)	0.51	1.76	(0.27-11.68)	0.56	0.51	(0.17-1.49)	0.22

PSC: Patient Safety Culture; CI: Confidence Interval.

^¶^Physicians do not usually work in a single section, but so are included in the physicians’ unit.

^‡^Results of the generalised linear mixed model (GLMM) using respondent-level data. The reference category was low-PSC units. The differences between hospitals were included in the GLMM as random effects.

^†^P <0.05.

## Discussion

Units in the hospitals were categorised into high-PSC units or low-PSC units. The percent positive scores for all sub-dimensions were significantly higher in the high-PSC units than in the low-PSC units. The structure of PSC in each unit type was simple, and there were no clusters with partially good or bad sub-dimensions. The combined unit type of ‘Obstetrics and gynaecology ward, perinatal ward or NICU’ tended to be categorised as high-PSC units, whereas ‘Long-term care ward’, ‘Rehabilitation unit’ and ‘Administration unit’ tended to be categorised as low-PSC units. The key sub-dimensions for categorisation as a high-PSC unit differed by unit type. To better interpret the results and review possible measures, it may be necessary to take background factors, such as differences in professional composition and responsibilities, for every unit type into account.

‘Obstetrics and gynaecology ward, perinatal ward or NICU’ was the only high-PSC unit in which ‘Organisational learning - continuous improvement’ and ‘Hospital management support for patient safety’ were rated highly. PSC may have been enhanced in obstetrics and gynaecology and perinatology because the risk of litigation is high [11]. However, the findings of our study with regard to obstetrics and gynaecology wards were contrary to the findings of previous studies. According to the AHRQ 2012 Hospital Comparative Database, a database of AHRQ PSC surveys that includes data from 567,703 respondents from 1,128 hospitals in the United States, obstetrics units had moderate PSC [12]. Moreover, some studies reported low PSC in obstetrics and gynaecology units [13,14]. These conflicting results may be due to differences in the respondent populations. For example, in obstetrics units, nurses may have better PSC than physicians [15]. In our study, most of the respondents in the ‘Obstetrics and gynaecology ward, perinatal ward or NICU’ were nurses, and physicians were included in ‘Physicians’ unit’. Therefore, the high proportion of nurses in our study may have contributed to the evaluation of high PSC in the obstetrics area. A study found better PSC in NICUs than in adult ICUs, but further studies are needed to determine PSC in perinatal wards and NICUs [16].

In administration units, the culture of improvements based on event reporting may not be established because the percent positive scores of ‘Frequency of events reported’ and ‘Organisational learning - continuous improvement’ were the lowest, and the proportion of respondents who reported one or more events during the previous year was also the lowest among the unit types. The healthcare workers in these units may not have been given the opportunity to improve their PSC because their activities do not usually involve caring for patients. Therefore, there would be few adverse events relating to patient care that needed to be reported.

In the present study, communication and information exchange with other units was poor in rehabilitation units because the percent positive scores for ‘Teamwork across hospital units’ and ‘Hospital handoffs and transitions’ were the lowest. Close coordination with other units and professionals is important in rehabilitation units, and the AHRQ 2012 Hospital Comparative Database report showed that the average percent positive score across the sub-dimensions in rehabilitation units in the United States was the highest among all work units [12]. The reasons for these conflicting results between Japan and the United States should be investigated in future research.

In long-term care wards, outcome measures for PSC, such as ‘Patient safety grade’ and ‘Overall perceptions of patient safety’, were the lowest among all unit types. Percent positive scores of PSC in nursing homes are lower than that in acute care hospitals [17,18]. In the United States, the reasons for this have been attributed to low staffing levels and a high rate of staff turnover in nursing homes [19-22]. Staffing issues in nursing homes reduce the quality of care because the continuity of care is lost, there is an increase in the number of unskilled workers, it is difficult to establish standards of care and there is an increased workload on the remaining staff [19-21]. In the present study, non-medical care workers accounted for half of the healthcare workers in long-term care wards, and there was also a high turnover rate because of low wages or poor working conditions [23-25]. In the United States, a high staffing level of registered nurses was associated with high PSC because the interactional norms and many of the organisational norms in nursing homes are highly influenced by nurses [26,27]. In long-term care wards, a reduction in the turnover of non-medical care workers and an increase in the staffing level of registered nurses could help to develop better personal relationships among the healthcare workers and improve ‘Communication openness’. In addition, ‘Supervisor/manager expectations and actions promoting safety’ and ‘Organizational learning - continuous improvement’ might also relate to the categorisation as high-PSC unit, but the confidence intervals of ORs for those two sub-dimensions were too large to confirm the relationships.

In ‘Critical care centre, ICU or CCU’, the ‘Staffing’ might relate to the categorisation as high-PSC unit. Sufficient resources might be required to control the higher level of intrinsic hazard, complexity, variety or rapidity of work in those areas and to maintain the PSC. The AHRQ 2012 Hospital Comparative Database report showed that the average percent positive score across the sub-dimensions in emergency departments in the United States was the lowest among all work units [12]. In the United States, some studies also reported poor PSC at emergency departments or ICUs although our study showed moderate PSC [8,9]. Reasons of the deference between the United States and Japan should be investigated in future research.

The key sub-dimensions that determined whether a unit was categorised as a high-PSC unit or a low-PSC unit differed by unit type. Further research is needed to identify the factors contributing to low PSC and to establish improvements tailored to each work unit type.

### Limitations

This study had a cross-sectional design, and causation cannot be established. In addition, the hospitals that participated in this study may not be representative of all hospitals in Japan. The actual patient safety level for each unit was not clearly identified because PSC was assessed subjectively. Some of our results may have limited statistical power because the numbers of some unit types were relatively small. Finally, the findings of this study may not be generalised for other countries because other countries may have a different PSC structure [6].

## Conclusions

Our study findings suggest that PSC scores in hospitals in Japan depend on the unit type. There is a high PSC in ‘Obstetrics and gynaecology ward, perinatal ward or NICU’, but a low PSC in ‘Long-term care ward’, ‘Rehabilitation unit’ and ‘Administration unit’. Factors contributing to low PSC need to be further investigated so appropriate measures to improve PSC can be developed and initiated.

## Additional files

Additional file 1:
**List of participating hospitals.**
Additional file 2:
**Professional composition of each unit type.**
Additional file 3:
**Percent positive scores of 12 sub-dimensions and 2 outcome measures for PSC.**

## Abbreviations

PSC: Patient safety culture
GLMM: Generalised linear mixed model
ORs: Odds ratios
HSOPSC: Hospital Survey of Patient Safety Culture
AHRQ: Agency for Healthcare Research and Quality
NICU: Neonatal intensive care units
